# Supplementary material for: Longitudinal Analysis of 1α,25-dihidroxyvitamin D3 and Homocysteine Changes in Colorectal Cancer
Source: Cancers (Basel). 2022 Jan 28;14(3):658. doi: 10.3390/cancers14030658 (PMC8833406; doi:10.3390/cancers14030658)
Supplement: Supplementary file 1 [file cancers-14-00658-s001.zip › cancers-1542612-supplementary.pdf]

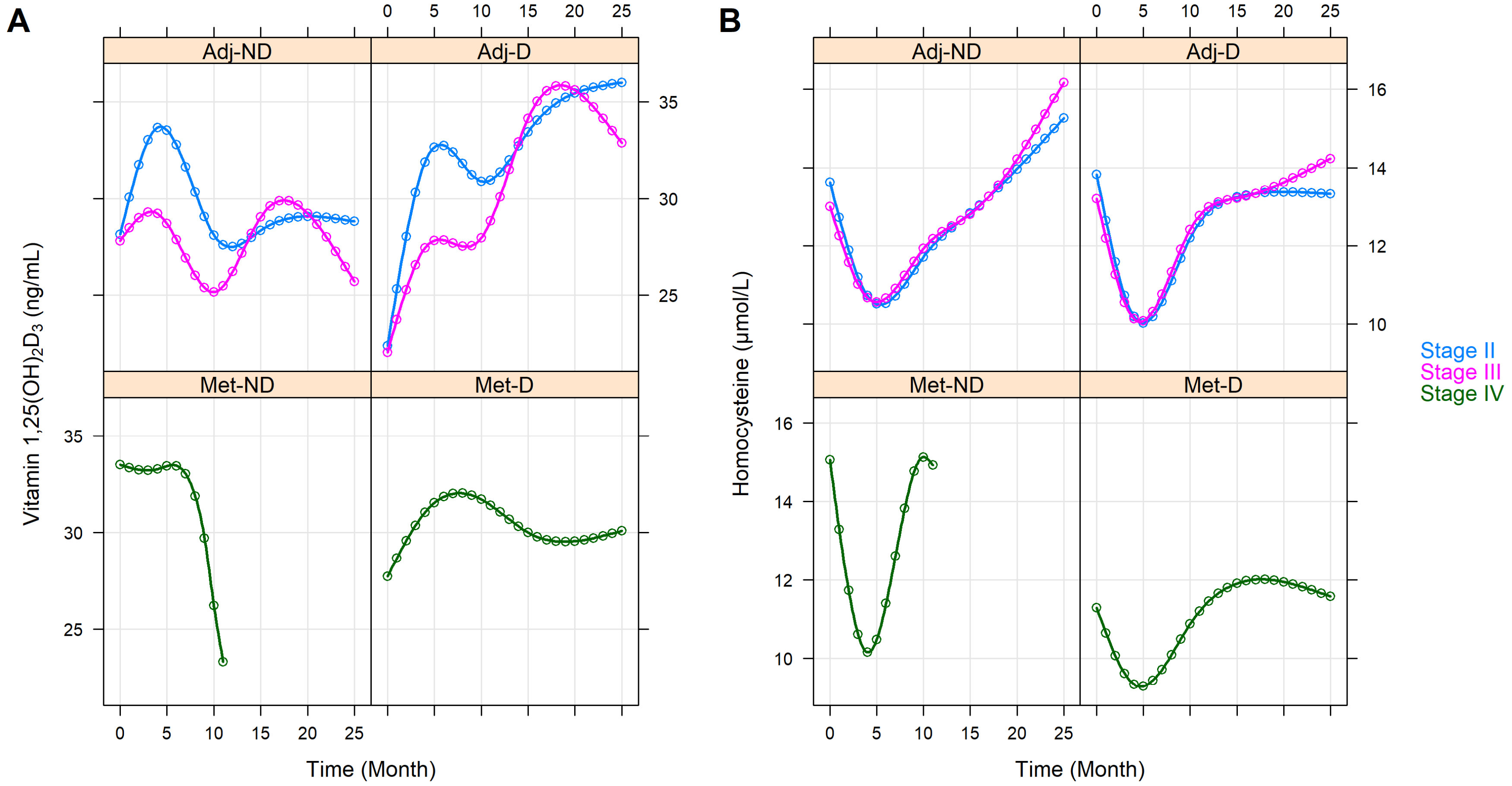


**Figure S1.** Predictions of average longitudinal changes in vitamin 1,25$(OH)_2D_3$ (**A**) and homocysteine (**B**) level of the study groups throughout the first 25 months of the observation time, stratified by AJCC staging [22]. Adj-ND: patients without metastasis and no vitamin $D_3$ supplementation; Adj-D: patients without metastasis with vitamin $D_3$ supplementation; Met-ND: patients with metastasis and no vitamin $D_3$ supplementation; Met-D: patients with metastasis with vitamin $D_3$ supplementation.

[22] Jessup, J.; Goldberg, R.; Asare, E.; Benson, A.; Brierley, J.; Chang, G.; Chen, V.; Compton, C.; De Nardi, P.; Goodman, K., et al. Colon and Rectum. In *AJCC Cancer Staging Manual (8th Edition)*, Amin, M., Edge, S., Greene, F., Byrd, D., Brookland, R., Washington, M., Gershenwald, J., Compton, C., Hess, K., Sullivan, D., et al., Eds. Springer International Publishing: Chicago, IL, USA, **2018**; pp. 251-274.

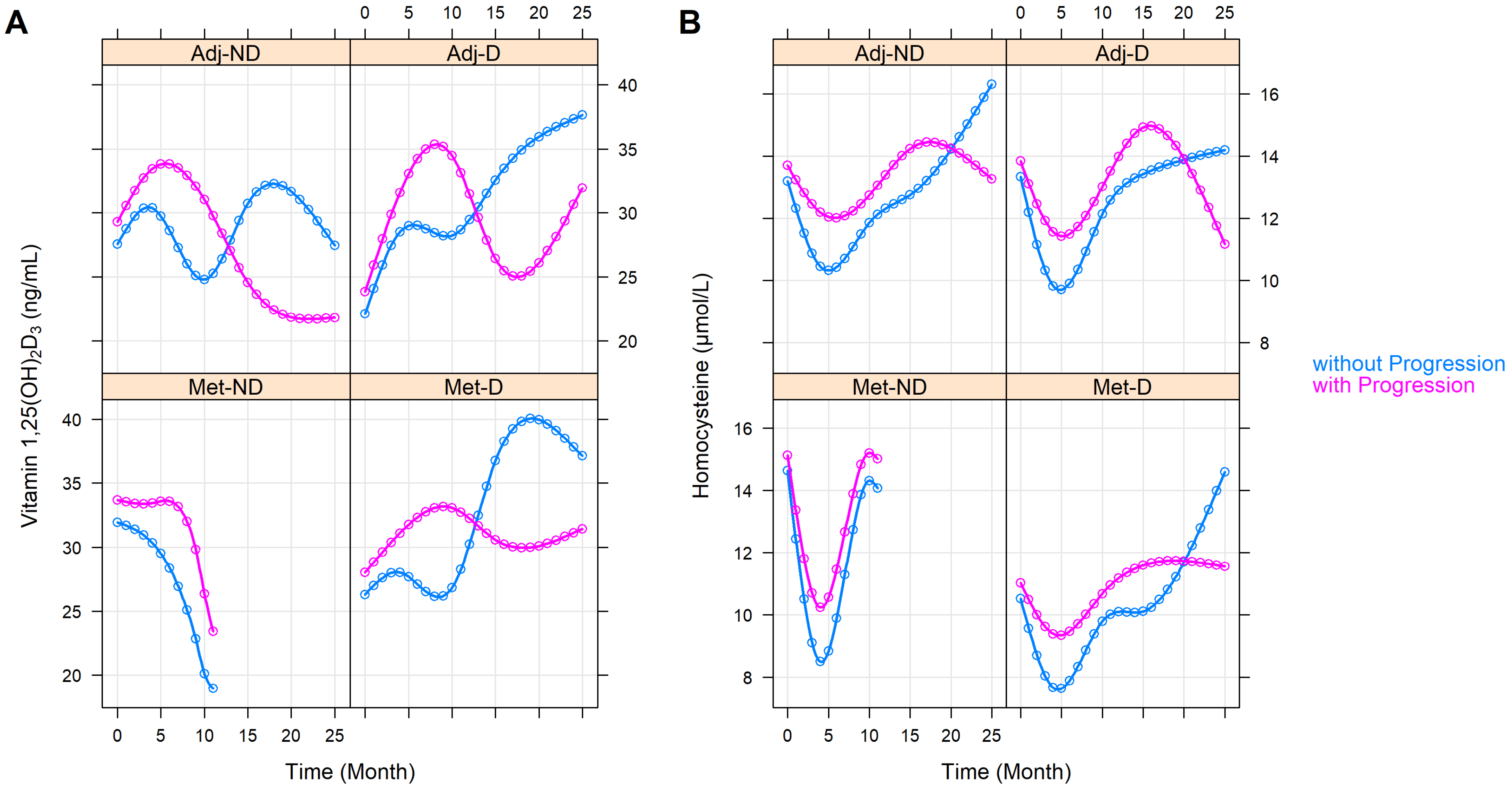


**Figure S2.** Predictions of average longitudinal changes in vitamin 1,25(OH)$_2$D$_3$ (**A**) and homocysteine (**B**) level of the study groups throughout the first 25 months of the observation time, stratified by the occurrence of disease progression. Adj-ND: patients without metastasis and no vitamin D$_3$ supplementation; Adj-D: patients without metastasis with vitamin D$_3$ supplementation; Met-ND: patients with metastasis and no vitamin D$_3$ supplementation; Met-D: patients with metastasis with vitamin D$_3$ supplementation.

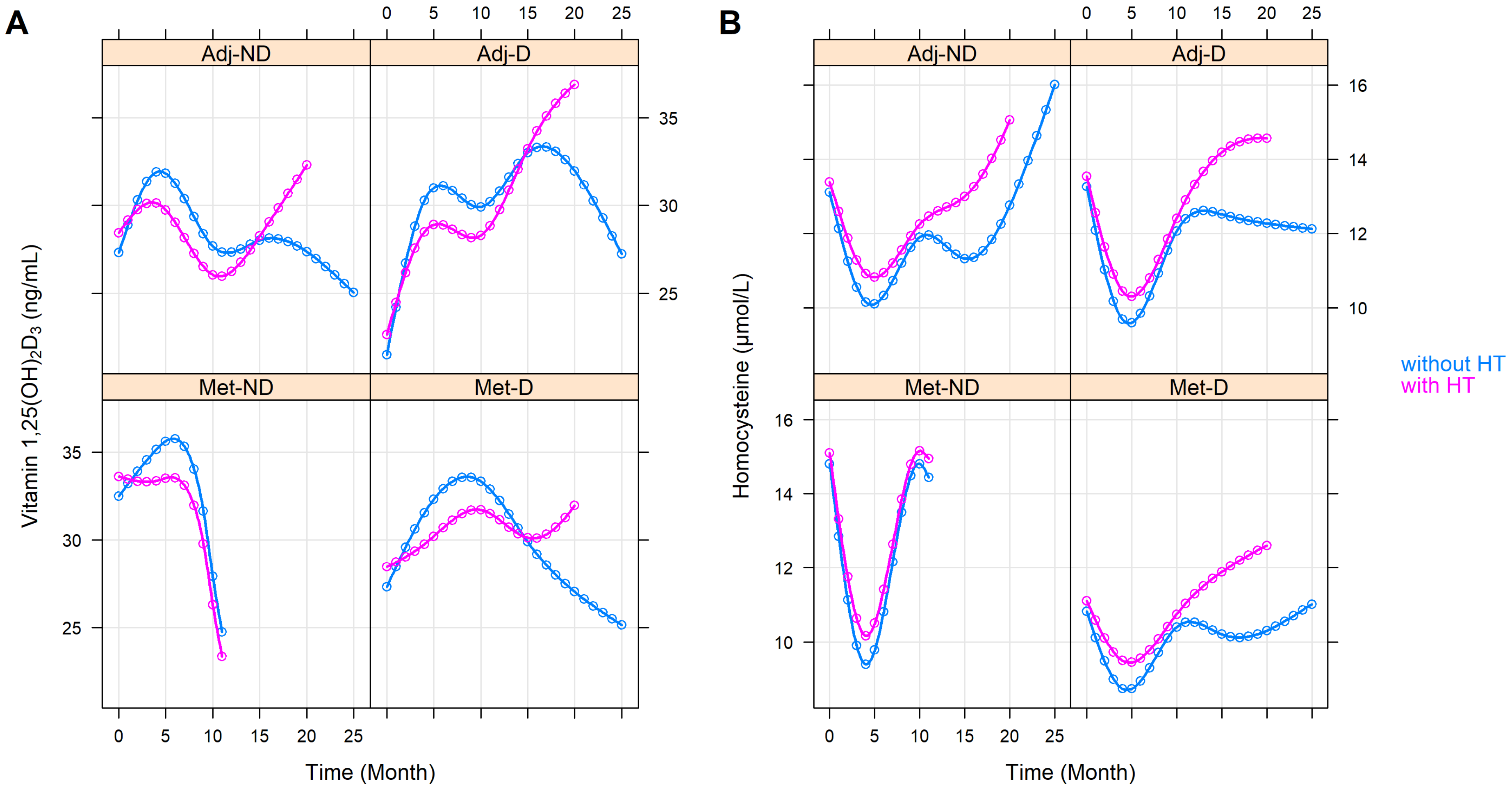


**Figure S3.** Predictions of average longitudinal changes in vitamin 1,25(OH)$_2$D$_3$ (**A**) and homocysteine (**B**) level of the study groups throughout the first 25 months of the observation time, stratified by the presence of hypertension (HT). Adj-ND: patients without metastasis and no vitamin D$_3$ supplementation; Adj-D: patients without metastasis with vitamin D$_3$ supplementation; Met-ND: patients with metastasis and no vitamin D$_3$ supplementation; Met-D: patients with metastasis with vitamin D$_3$ supplementation.

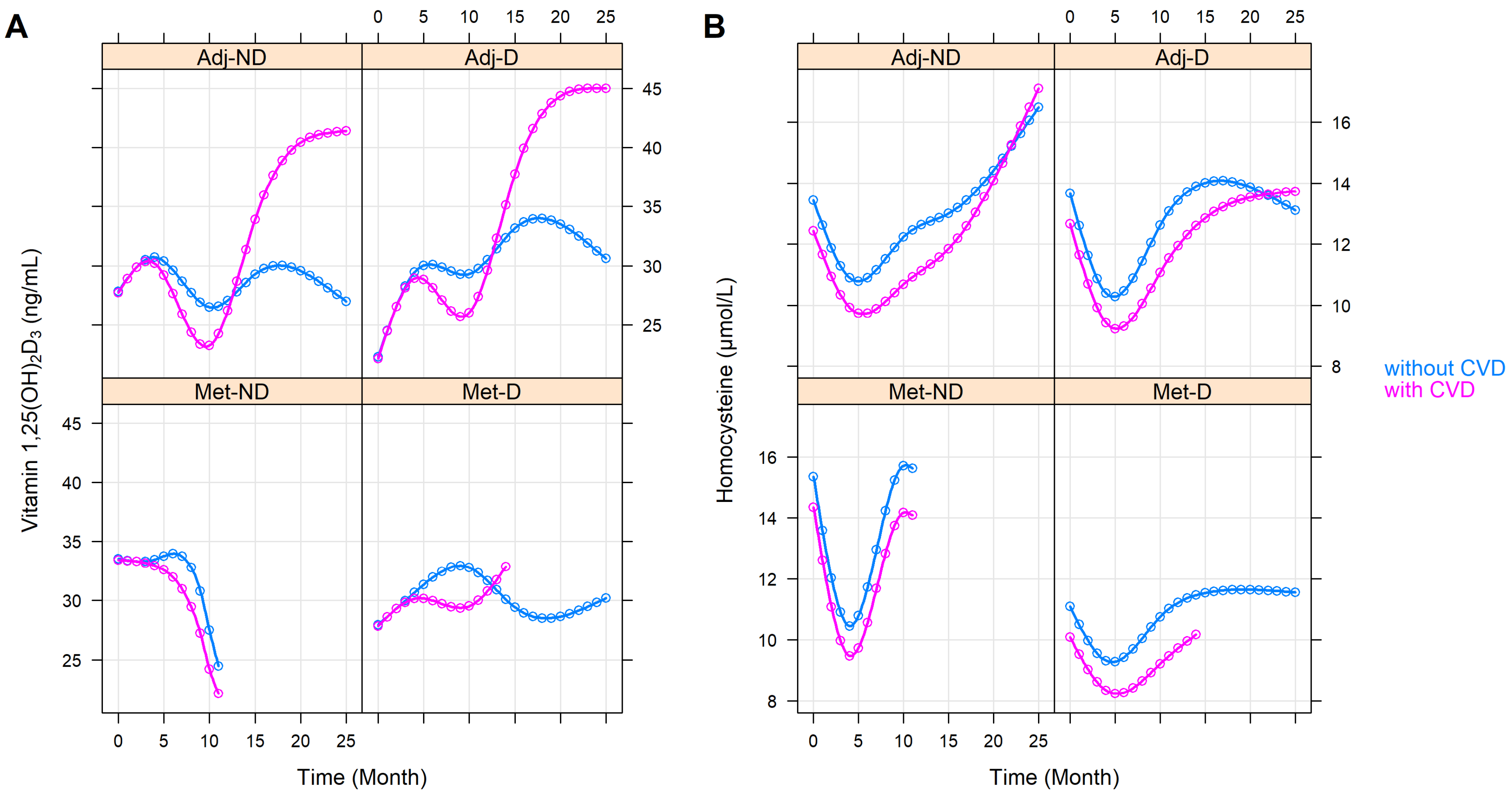


**Figure S4.** Predictions of average longitudinal changes in vitamin 1,25(OH)$_2$D$_3$ (**A**) and homocysteine (**B**) level of the study groups throughout the first 25 months of the observation time, stratified by the presence of cardiovascular diseases (CVD) excluding previous major cardiovascular events (*e.g.* stroke, infarct, etc.) and hypertension. Adj-ND: patients without metastasis and no vitamin D$_3$ supplementation; Adj-D: patients without metastasis with vitamin D$_3$ supplementation; Met-ND: patients with metastasis and no vitamin D$_3$ supplementation; Met-D: patients with metastasis with vitamin D$_3$ supplementation.

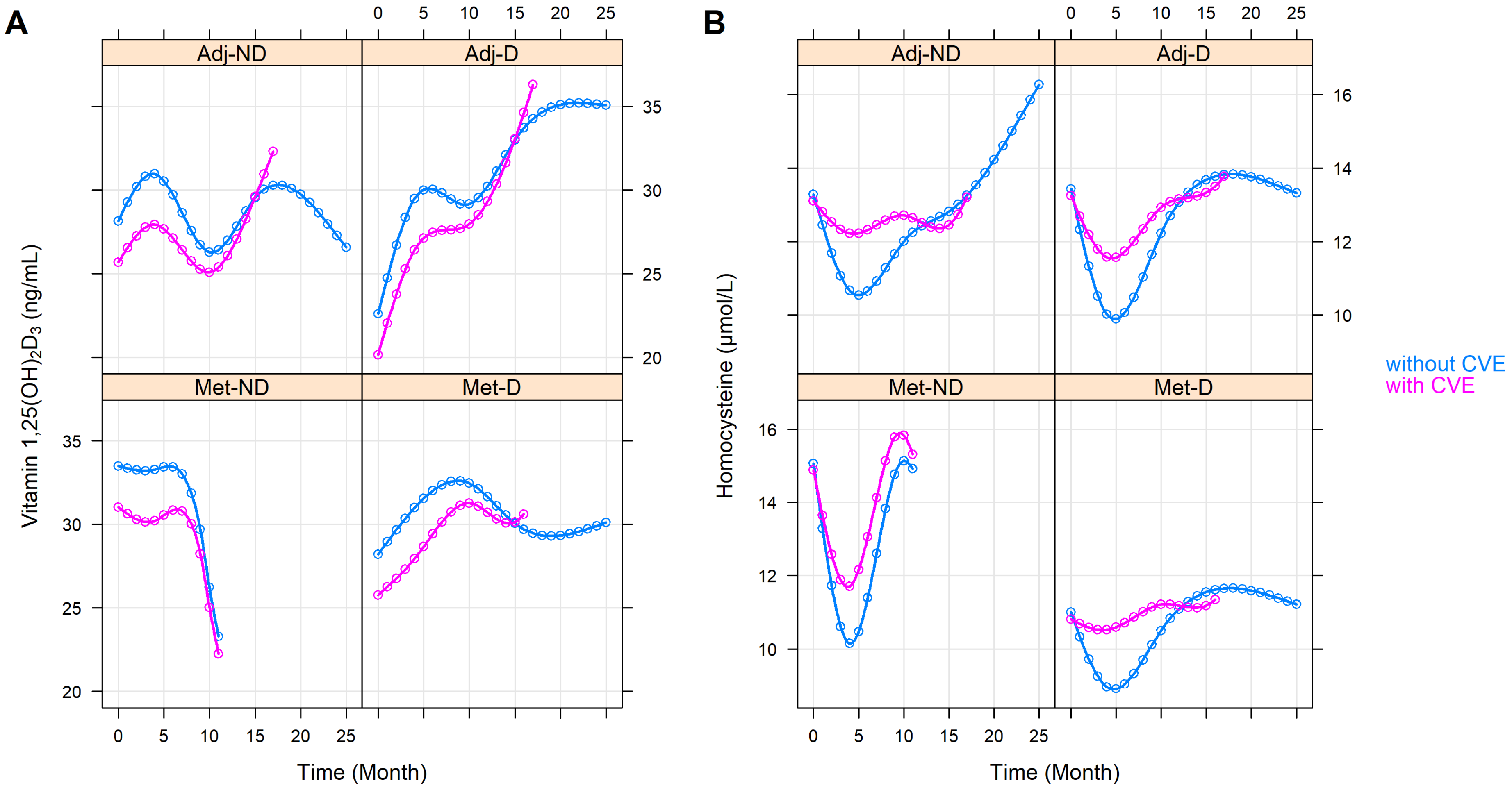


**Figure S5.** Predictions of average longitudinal changes in vitamin 1,25(OH)$_2$D$_3$ (**A**) and homocysteine (**B**) level of the study groups throughout the first 25 months of the observation time, stratified by the presence of previous cardiovascular events (CVE), such as stroke, myocardial infarct, *etc*. Adj-ND: patients without metastasis and no vitamin D$_3$ supplementation; Adj-D: patients without metastasis with vitamin D$_3$ supplementation; Met-ND: patients with metastasis and no vitamin D$_3$ supplementation; Met-D: patients with metastasis with vitamin D$_3$ supplementation.

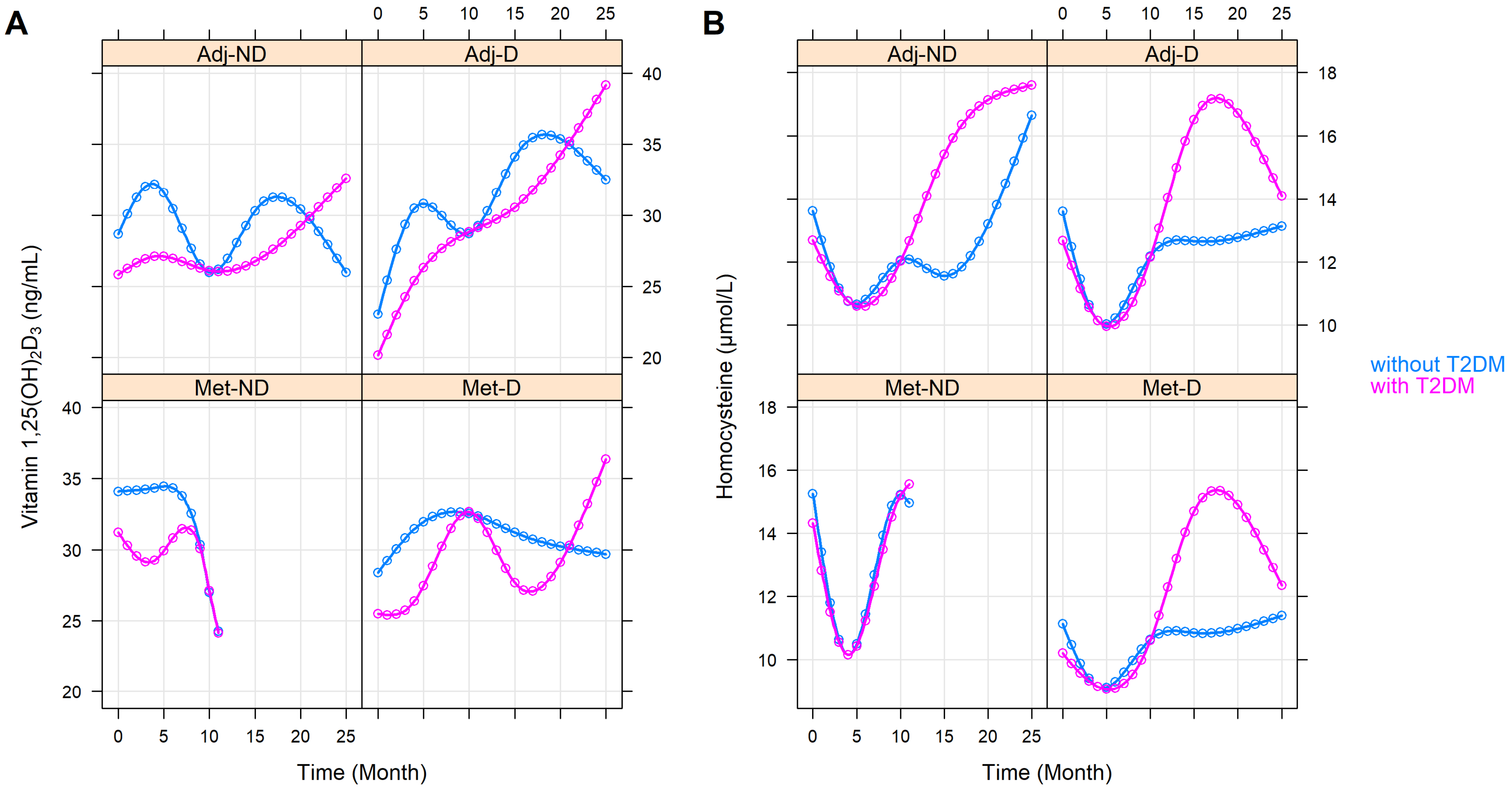


**Figure S6.** Predictions of average longitudinal changes in vitamin 1,25(OH)$_2$D$_3$ (**A**) and homocysteine (**B**) levels of the study groups throughout the first 25 months of the observation time, stratified by the presence of type 2 diabetes mellitus (T2DM). Adj-ND: patients without metastasis and no vitamin D$_3$ supplementation; Adj-D: patients without metastasis with vitamin D$_3$ supplementation; Met-ND: patients with metastasis and no vitamin D$_3$ supplementation; Met-D: patients with metastasis with vitamin D$_3$ supplementation.

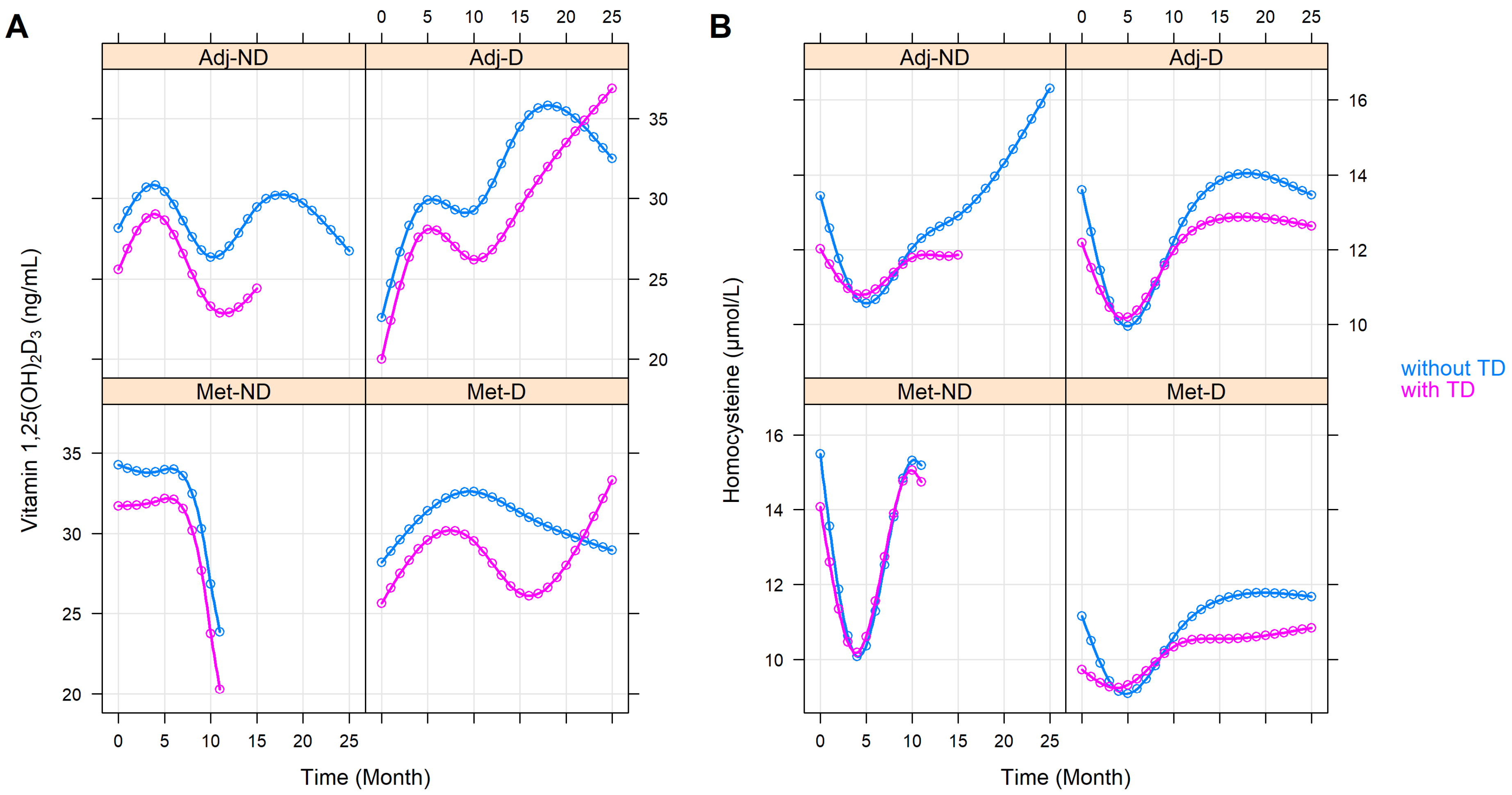


**Figure S7.** Predictions of average longitudinal changes in vitamin 1,25(OH)$_2$D$_3$ (**A**) and homocysteine (**B**) level of the study groups throughout the first 25 months of the observation time, stratified by the presence of any thyroid diseases (TD). ***Note: patients who were not in an euthyroid state were excluded from the study.*** Adj-ND: patients without metastasis and no vitamin D$_3$ supplementation; Adj-D: patients without metastasis with vitamin D$_3$ supplementation; Met-ND: patients with metastasis and no vitamin D$_3$ supplementation; Met-D: patients with metastasis with vitamin D$_3$ supplementation.
